# HIV Status and Other Risk Factors for Prevalent and Incident Sexually Transmitted Infection during Pregnancy (2000-2014)

**DOI:** 10.1155/2019/6584101

**Published:** 2019-04-01

**Authors:** Jodie Dionne-Odom, Michelle J. Khan, Victoria C. Jauk, Jeff Szychowski, Dustin M. Long, Suzanne Wallace, Cherry Neely, Karen Fry, Jeanne Marrazzo, Marilyn Crain, Alan T. N. Tita

**Affiliations:** ^1^Department of Medicine, Division of Infectious Diseases, University of Alabama at Birmingham, Birmingham, Alabama, USA; ^2^Departments of Obstetrics and Gynecology and Adult and Family Medicine, Kaiser Permanente, San Leandro, California, USA; ^3^Department of Obstetrics and Gynecology, Division of Maternal-Fetal Medicine, Center for Women's Reproductive Health, University of Alabama at Birmingham, Birmingham, Alabama, USA; ^4^Department of Biostatistics, University of Alabama at Birmingham School of Public Health, Birmingham, Alabama, USA; ^5^Department of Pediatrics, Division of Infectious Diseases, University of Alabama at Birmingham, Birmingham, Alabama, USA

## Abstract

**Background:**

Sexually transmitted infections (STIs) are associated with adverse birth outcomes. Current prenatal STI screening guidelines define “risk” without explicit consideration of HIV status. Our objective was to test the hypothesis that HIV status is associated with bacterial STI in pregnant women.

**Methods:**

We designed a retrospective cohort study to identify pregnant women with HIV who delivered at our facility during 2000-2014. HIV+ women were compared to HIV- women with matching by year of delivery. Logistic regression was used to model adjusted odds of prevalent and incident STI. Prevalent STI was defined as chlamydia (CT), gonorrhea (GC), syphilis, or trichomoniasis detected on an initial prenatal screening test and incident STI as a newly positive result following a negative prenatal test.

**Results:**

The cohort included 432 women, 210 HIV+ and 222 HIV-. Most pregnant women were screened for STI (92% of HIV+ women and 74% of HIV- women). STI rates were high and particularly elevated in HIV+ women: 29% vs 18% (p=0.02), for prevalent STI and 11% vs 2% (p<0.001) for incident STI. Risk factors for prevalent STI were as follows: HIV status (aOR 3.0, CI: 1.4-6.4), Black race (aOR 2.7, 95% CI: 1.1-6.6), and more recent delivery (2007-2014 compared to 2000-2006) (aOR 2.3, CI: 1.1-4.7). HIV status was an independent risk factor for incident STI (aOR 7.2, CI: 2.1-25.0).

**Conclusion:**

Pregnant women who delivered in our center had high STI rates. Since HIV infection was independently associated with prevalent and incident STI, prenatal screening guidelines may need to incorporate HIV status as a high-risk group for repeat testing.

## 1. Introduction

More than 2 million cases of sexually transmitted infections (STI) in the United States were reported to the U.S. Centers for Disease Control (CDC) in 2017 [1]. Young adults are disproportionately impacted by STI, with important implications for women of reproductive age. Congenital syphilis rates increased 153% since 2013 to 23 cases per 100,000 live births as primary and secondary syphilis rates rise in women [1]. STI and HIV rates are elevated in the Southeastern region [2, 3]. Pregnancy desires in women living with HIV are similar to the general population and successful Prevention of Mother to Child Transmission (MTCT) interventions have reduced vertical HIV transmission rates from 25% to <1% [4, 5].

The most common and curable STIs in pregnancy are caused by* Chlamydia trachomatis* (CT),* Neisseria gonorrhoeae* (GC),* Treponema pallidum* (syphilis), and* Trichomonas vaginalis* (TV). Independently, both pregnancy and HIV infection can increase susceptibility to infection. In pregnancy, this increased susceptibility to STI has been attributed to various factors: anatomic (cervical ectropion), immunologic (reduction in humoral and cell-mediated immunity) and behavioral (new or risky sexual partners) but it is not known if pregnant women with HIV are at additional risk of STI [6]. Adverse outcomes associated with STI in pregnancy include preterm delivery (PTD), low birthweight and stillbirth, and HIV/STI coinfection is associated with a 2-fold increase in PTD and vertical HIV transmission [7–12]. Factors associated with increased STI prevalence in pregnant women include the following: age <25, Black race, and low socioeconomic status [1, 13, 14]. Although HIV infection in adults may be a marker of risk behavior, many women with HIV in care report few or no current STI risk behaviors [15].

Screening for asymptomatic STI during routine prenatal care has been a longstanding and cost-effective recommendation by the American College of Obstetricians and Gynecologists (ACOG), CDC, and US Preventive Services Task Force (USPSTF) [16–18]. Bacterial STI screening guidelines in pregnancy do not explicitly take maternal HIV status into account. Screening for gonorrhea is recommended at the first prenatal visit if age <25 or older women with risk factors, while universal screening is recommended for syphilis and HIV. In 2017, ACOG recommended universal chlamydia screening in pregnancy but CDC continues to recommend only risk based screening for pregnant women above age 25. Trichomoniasis screening is only recommended in women with HIV [16]. Repeat HIV testing in the 3^rd^ trimester is recommended for women at risk, including those diagnosed with STI during pregnancy [16]. CDC and USPSTF guidelines for CT/GC screening in pregnancy define risk in terms of sex partner characteristics (new, nonmonogamous or with known STI) [16, 19].

In order to document rates and risk factors for STI infection during pregnancy in a high morbidity region of the US, we conducted a retrospective cohort analysis of prevalent and incident STI among pregnant women with and without HIV who delivered at our center during a 15-year period. Our main hypothesis was that HIV status would be independently associated with prevalent and incident STI during pregnancy.

## 2. Materials and Methods

### 2.1. Study Design and Population

A retrospective matched cohort design was used to study women with singleton pregnancies followed in the Obstetrics Clinics who subsequently delivered at the University of Alabama at Birmingham (UAB) Hospital between January 1^st^, 2000, and December 31^st^, 2014. UAB Women and Infants Center is the largest referral hospital in the state with provision of specialty obstetric clinical care and approximately 4000 deliveries annually. Women who deliver at UAB Hospital include patients seen in outlying public health clinics, UAB high-risk obstetrics clinics, and by general academic obstetric providers. The cohort was comprised of two groups who received prenatal care in our system: women with confirmed HIV infection and a comparison group of randomly selected, HIV-uninfected women followed in the same obstetrics clinics. Women in both groups were matched by year of delivery. If a study participant had more than one pregnancy during the study period, only information from the first pregnancy was included. Clinical and laboratory data (HIV viral load, CD4 count, STI test results) were abstracted from the electronic medical record. Information about substance abuse (marijuana and illicit drugs) and tobacco use prior to pregnancy was collected by self-report. Illicit drug use during pregnancy was according to self-report or laboratory testing and psychiatric disease was defined as depression, anxiety, bipolar disorder or schizophrenia in the medical record.

### 2.2. STI Diagnostic Testing in Pregnancy

Testing for CT and GC was by culture at the Alabama Department of Health State Laboratory between 2000 and 2006 and by molecular diagnostic testing at the UAB OBGYN Research and Diagnostic Laboratory between 2007 and 2014. This timing was used to split the cohort into two study periods. Automated systems used DNA PCR to detect* Chlamydia trachomatis* and* Neisseria gonorrhoeae *on specimens collected at cervical, vaginal and urinary sites (Roche Amplicor in 2007-2012, Roche COBAS 4800 in 2013-2014; Pleasanton, California).

Syphilis testing was by RPR screening performed on site with confirmatory treponemal testing by* Treponema pallidum* particle agglutination assay (TPPA) at the Alabama Department of Public Health Laboratory. The reverse testing algorithm (starting with a treponemal EIA screen) was adopted at the end of the study period in November 2014 [20]. Positive treponemal and nontreponemal syphilis tests were required for syphilis cases.

Diagnostic testing for* Trichomonas vaginalis* was performed by wet mount microscopy of vaginal fluid.

### 2.3. Other Diagnostic Testing in Pregnancy

Non-STI test results with relevance to perinatal management were also abstracted from the medical record. Hepatitis B infection was defined by the presence of HBV surface antigen (HBSAg+) in serum, hepatitis C infection by the presence of HCV antibody (HCV Ab+), vaginal candidiasis was a clinical diagnosis, and* Streptococcus agalactiae* (GBS) colonization was detected by culture of an anogenital swab collected by the provider at 35-37 weeks gestational age. History of genital herpes simplex virus (HSV) infection was according to self-report.

### 2.4. Statistical Analysis

The two primary outcomes were prevalent and incident STI with the analysis restricted to women who were screened for STI during pregnancy. Prevalent STI was defined as the detection of chlamydia, gonorrhea, syphilis, and/or trichomonas on the initial screening test. Incident STI in pregnancy was defined as a newly positive diagnostic test for chlamydia, gonorrhea, syphilis, and/or trichomoniasis infection following a negative test earlier in pregnancy. Overall rates and 95% confidence intervals are presented. The denominator varied for individual STIs based on the number of women screened. All women were considered at risk of incident STI, irrespective of the presence of prevalent STI.

Patient characteristics including demographics, substance use, medical comorbidities (such as hepatitis), and study time period were compared between women with and without HIV. Differences in characteristics between the two groups of pregnant women were compared using the chi-square test of association or Fisher's exact test, as appropriate, for categorical variables or the Student's t-test for continuous variables.

Logistic regression models were used to evaluate potential risk factors for STI. Variables identified as statistically significant in initial bivariate analyses (p<0.05) or defined a priori were subsequently evaluated, one at a time, in logistic regression models. Variables with high levels of missing data (such as education) were not included in the models. Next, these variables were included as covariates in larger multivariable logistic regression models for prevalent and incident STI. Odds ratios (OR) and 95% confidence intervals (CI) were evaluated. A parsimonious regression model was determined using backward elimination (using p>0.05). The characteristic with the largest p value was eliminated in each iteration until reaching a final, reduced model where the remaining covariates were statistically significant at <0.05 level. Analyses were performed with SAS v 9.4. (SAS Institute, Cary, NC).

### 2.5. Ethics

The study was approved by the Institutional Review Board at the University of Alabama at Birmingham with a waiver for informed consent.

## 3. Results

### 3.1. Characteristics of Women with HIV

There were 432 deliveries in this cohort of women in Alabama between 2000 and 2014: 210 were women living with HIV and 222 in women without HIV infection selected randomly from the clinic population and matched by year of delivery. Participant characteristics stratified by HIV status are shown in Table 1. Age, race, and marital status differed by HIV status but parity and socioeconomic status, as measured by insurance type and education level were similar in both groups. The median CD4 count at the initial antepartum visit was at the low end of the normal range (410 cells/mm^3^; IQR 294-523) and the median HIV viral load at the initial prenatal visit was 4160 copies/mL. At the initial visit, one in three women (33.1%, 52/157) had viral load <1000 copies/mL and only 1.3% had an undetectable viral load (<50 copies/mL). The timing of this HIV viral load testing was variable (40.6%  1^st^ trimester, 50.3%  2^nd^ trimester, 9.1%  3^rd^ trimester). Follow up viral load testing at delivery was available for a subset of 92 women who delivered between 2009 and 2013; 92.4% (85/92) had HIV viral load <1000 copies/mL.

### 3.2. Prevalent and Incident STI by HIV Status

Table 2 shows the frequency of prevalent and incident chlamydia, gonorrhea, syphilis, trichomoniasis, and other infections among women screened for STI in pregnancy. Pregnant women with HIV were more likely to be screened for STI (193/210 or 91.9%) compared to women without HIV (165/222 or 74.3%). In the study population overall, 24% (95% CI 20%-28%) had prevalent STI and 7% (95% CI 4%-9%) had incident STI. Women with HIV accounted for 65.1% (56/86) of prevalent cases and 87.5% (21/24) of incident STI cases. Of 71 prevalent infections with the date of test available, 46.5% were detected in the first trimester, 23.9% in the 2^nd^ trimester and 29.6% in the 3^rd^ trimester. This may reflect the time of entry to care. Prevalent and incident STI rates were significantly higher in women living with HIV compared to HIV-uninfected women (29.0% vs 18.2%, respectively, (p=0.02)) for prevalent STI and 10.9% vs 1.8% (p<0.001) for incident STI). Individually, chlamydia, gonorrhea and trichomoniasis were more common in the HIV-infected group but differences were not statistically significant. Among the 27 incident STIs detected, chlamydia (55.6%) and trichomonas (26.0%) were most common. There was no difference in the proportion of women with more than 1 prevalent or incident STI during pregnancy by HIV status. Pregnant women with HIV were also more likely to have other infections: active hepatitis B (4.9% vs 0.6%), anogenital GBS colonization (32.8% vs 18.0%), and HSV (23.3 vs 8.8%) and to have vulvovaginal candidiasis (16.1% vs 6.8%). HIV-infected women with prevalent and incident STI had similar or higher median CD4 counts compared to HIV-infected women without STI (data not shown). Hepatitis C (1.7% vs 1.9%) and bacterial vaginosis rates were similar in both groups (3.6% vs 2.5%).

### 3.3. Models for Prevalent and Incident STI in Pregnancy

Risk factors for STI during pregnancy are shown in Table 3. In the unadjusted model for prevalent STI, HIV infection, Black race, public/no insurance compared to private, single marital status, and more recent delivery were predictive. In the adjusted model for prevalent STI, three independent predictors were identified: HIV infection (aOR 3.0, CI 1.4-6.4), Black race (aOR 2.7, 95% CI 1.1-6.6), and delivery after 2007 (aOR 2.3, CI 1.1-4.7). In the unadjusted model for incident STI, significant predictors were HIV infection, Black race, delivery after 2007, psychiatric disease, and age. In the adjusted model for incident STI, HIV status (aOR 7.2, CI 2.1-25.0) and delivery after 2007 (aOR 8.6, CI 2.0-37.8) were associated. As the amount of missing data could bring into question the validity of our findings, sensitivity analyses using multiple imputation methods were performed for the adjusted models and the results were similar (not shown).

## 4. Discussion

Chlamydia, gonorrhea, syphilis, and/or trichomonas were detected in one in four pregnant women in Alabama during the study period. One in 12 women had more than one STI diagnosed during pregnancy. Pregnant women living with HIV were at particular risk of STI acquisition with 3-7-fold increased odds compared to pregnant women without HIV. Elevated STI rates occurred despite the fact that women with HIV were older (younger age is one of the strongest predictors of STI acquisition risk in women) and not significantly immunocompromised.

Population-based, nationally representative data on STI prevalence and predictors of infection during pregnancy in the US are few [1]. In Baltimore in 1996-2002, STI prevalence (CT/GC/syphilis/TV) among 730 pregnant women seen in STD clinics was 43% [11]. In a recent study of birth outcomes among 2389 HIV-infected pregnant women followed in US cohort studies, 28%-39% had STI in pregnancy [21]. These STI rates are comparable to our study. In contrast, in a survey of 13,000 women from 5 US states as part of the Pregnancy Risk Assessment Monitoring System (PRAMS), self-reported STI prevalence (CT/GC/syphilis/TV) during recent pregnancy was 3.3% [13]. Self-reported STI survey data likely underestimated true infection prevalence. This PRAMS study identified STI predictors in pregnancy that are similar to nonpregnant women: (Black race, age <25, single marital status, and low socioeconomic status) but maternal HIV status was not included [13].

The current analysis shows a strong and significant association between HIV status and STI acquisition during pregnancy. This finding cannot be entirely explained by ascertainment bias due to higher STI screening rates in women with HIV compared to women without HIV (92% vs 74%). Both groups had high screening rates and high positivity rates. Although domestic studies are few and the question of STI susceptibility in HIV-infected women remains, syphilis, chlamydia, and gonorrhea infections are common in HIV-infected pregnant women globally where they contribute to high rates of adverse birth outcomes [7, 8, 12, 22]. Recent evidence from HIV serodiscordant couples in Africa suggests that the 2^nd^ and 3^rd^ trimester and postpartum periods are particularly risky in terms of HIV acquisition compared to the 1^st^ trimester period [23]. This may be relevant for STI acquisition as well, but additional studies to investigate the underlying biologic mechanisms of acquisition risk in pregnancy are needed.

If larger (ideally, prospective) studies confirm that HIV is independently associated with increased risk of STI acquisition, it will be useful to determine the attributable fraction of individual risk factors [24–29]. Measures of socioeconomic status (marital status, insurance status) dropped out of our prediction model for prevalent STI after adjustment. Also, substance abuse and psychiatric disease were not associated with prevalent STI, but our methods of ascertainment for these comorbid conditions were crude. Drug use in women with HIV also remains a serious issue in Alabama and the US, but these rates have declined over time in one longitudinal HIV cohort study [30]. Pregnant women in Baltimore reported fewer STI risk behaviors than nonpregnant women, yet STI rates were similar in both groups [31]. It remains unclear whether pregnancy itself increases risk of STI acquisition, (as it does for HIV acquisition) [28].

Black race was independently associated with prevalent STI in our model. This mirrors high STI rates among Black females in the USA [1]. The reasons for this association are not clear but likely relates to sexual networks and partner characteristics [32]. Younger age also predicted STI in pregnancy—a factor that tracks well with national epidemiology of STI in which women ages 18-25 are at highest risk [1]. Although it is possible that STI rates increased over time in our study population since gonorrhea and syphilis rates in women have been increasing since 2012 (and since 1999 for chlamydia), the association between STI and more recent delivery (2007-2014, compared to 2000-2006) in our model is likely artifactual due to the availability of nucleic acid amplification testing for chlamydia and gonorrhea after 2007 [1]. Molecular testing is much more sensitive compared to older, culture-based, technology. Emerging data also show an association between the drug use epidemic in the US and increasing STI rates in women [33–35].

Elevated STI rates in pregnancy demand additional attention to screening practices since 80-90% of infections are asymptomatic and many women are not aware that they are at risk [13]. Although more than 285,000 women are living with HIV in the United States, current prenatal STI screening guidelines do not incorporate HIV status [36]. Compliance with these guidelines among commercially insured pregnant women in the United States averages 85-98% for syphilis and 69-83% for chlamydia and gonorrhea; rates that are comparable to the 83% testing rate in the current study [37]. More attention is needed to ensure high rates of screening at the initial visit and repeat testing among women at risk. For women with HIV, testing for hepatitis B (which can be sexually acquired) and GBS colonization (which is not an STI) continue to be important given available perinatal interventions.

## 5. Limitations and Strengths

Limitations of this retrospective cohort study include the sample size and our inability to adjust for all potential confounders. Heterogeneity was introduced by screening practice and protocols that varied by provider and time period. It is possible that the difference in STI rates according to HIV status is due to bias caused by differential screening rates but STI prevalence and incidence in both groups may be underestimates given the lack of universal screening. Study estimates may also underestimate infection prevalence since some women may have received STI testing or treatment at outside facilities that were not captured in our dataset. Data related to alcohol intake, drug use, and HSV was limited by self-report and social desirability bias, and the presence of symptoms, behavioral data and partner information were not available. Trichomoniasis was likely underestimated given the limited sensitivity of wet mount testing. Findings in this high-risk group in Alabama may not generalize to all pregnant women (+/- HIV) in the US or those without prenatal care. Study strengths include the longitudinal cohort, and electronic medical record data including pregnancy and HIV care visits. One particular strength was our ability to identify both prevalent and incident STI given longitudinal data.

The implications of this study are threefold. (1) STI rates in our setting are high among pregnant women with and without HIV infection, so screening recommendations should be followed closely. Highly sensitive and specific diagnostic molecular diagnostic testing is now available for most curable infections. (2) It may be necessary to explicitly add maternal HIV status as “high-risk” in terms of prenatal STI screening guidelines. (3) Providers caring for pregnant women should work closely with public health professionals to identify new strategies to avoid reinfection, such as expedited partner STI therapy (which is permissible in 41 states) and with pediatricians to ensure awareness of STI-exposed neonates. Finally, engagement in prenatal and HIV care is required to implement optimized prenatal STI screening practice and treatment of infection.

## 6. Conclusions

Pregnant women in our urban Alabama facility have high rates of chlamydia, gonorrhea, syphilis and trichomoniasis. HIV status was a strong and independent predictor of prevalent and incident STI. Optimized prenatal STI screening practice is necessary to improve related birth outcomes in vulnerable groups since infection is often asymptomatic and national STI rates continue to rise.

## Figures and Tables

**Table 1:** Characteristics of pregnant women by HIV status (n=432).

Characteristic	Total n (%) or mean (SD) n=432	HIV-infected n (%) or mean (SD) n=210	HIV-uninfected n (%) or mean (SD) n=222	p-value
*Sociodemographics*				
Maternal Age, mean (SD)	26.6 (6.2)	27.3 (5.8)	25.8 (6.5)	0.01
Maternal Age, categories				<0.001
<20	48/430 (11.2)	10/209 (4.8)	38/221 (17.2)	
20-29	254/430 (59.1)	127/209 (60.8)	127/221 (57.5)	
30-39	116/430 (27.0)	67/209 (32.1)	49/221 (22.2)	
40+	12/430 (2.8)	5/209 (2.4)	7/221 (3.2)	
Race				<0.001
Black	277/431 (64.3)	164/209 (78.5)	113/222 (50.9)	
White/Hispanic/Other	154/431 (35.7)	45/209 (21.5)	109/222 (49.1)	
Marital Status				0.02
Single*∗*	310/406 (76.4)	157/192 (81.8)	153/214 (71.5)	
Married	96/406 (23.7)	35/192 (18.2)	61/214 (28.5)	
Highest Level of Education				0.25
<12^th^ grade	86/210 (41.0)	36/98 (36.7)	50/112 (44.6)	
≥ 12^th^ grade	124/210 (59.1)	62/98 (63.3)	62/112 (55.4)	
Insurance Status				0.86
Public/Uninsured	366/431 (84.9)	179/210 (85.2)	187/221 (84.6)	
Private	65/431 (15.1)	31/210 (14.8)	34/221 (15.4)	
Parity				0.84
0	138/389 (35.5)	62/182 (34.1)	76/207 (36.7)	
1	121/389 (31.1)	57/182 (31.3)	64/207 (30.9)	
2+	130/389 (33.4)	63/182 (34.6)	67/207 (32.4)	
Year of Delivery				0.79
2000-2006	170/432 (39.4)	84/210 (40.0)	86/222 (38.7)	
2007-2014	262/432 (60.7)	126/210 (60.0)	136/222 (61.3)	

*Substance Use*				
Before Pregnancy (self-report)				
Tobacco	102/369 (27.6)	56/186 (30.1)	46/183 (25.1)	0.29
Marijuana	33/300 (11.0)	15/145 (10.3)	18/155 (11.6)	0.73
Cocaine/IVDA/Other*∗∗*	36/303 (11.9)	22/152 (14.5)	14/151 (9.3)	0.16
During Pregnancy (self-report or labs)				
Cocaine	11/278 (4)	10/140 (7.1)	1/138 (0.7)	<0.01
IV Drugs	4/271 (1.5)	1/131 (0.8)	3/140 (2.1)	0.62

*Comorbidities*				
Psychiatric Disease*∗∗∗*	78/385 (20.3)	56/206 (27.2)	22/179 (12.3)	<0.001
HIV Diagnosis	N/A		N/A	
During Current Pregnancy		39/110 (35.5)		
Prior to Current Pregnancy		71/110 (64.6)		
Median CD4 at Initial antepartum Visit (IQR) (cells/mm^3^)		410 (294-523)	N/A	
CD4 Category at Initial antepartum Visit (cells/mm^3^)				
<200	N/A	22/188 (11.7)	N/A	
201-350		47/188 (25.0)		
351-500		63/188 (33.5)		
>500		56/188 (29.8)		
HIV Viral Load at Initial antepartum Visit (copies/mL) *∗∗∗∗*	N/A	4160 (477-22,203)	N/A	

*∗* includes divorced, widowed, separated. *∗∗* Other includes prescription drugs, methadone, suboxone, methamphetamine, LSD, morphine, and “street drugs.” *∗∗∗* Depression, anxiety, bipolar disorder, and/or schizophrenia. *∗∗∗∗* Among 157 women with VL available.

**Table 2:** Infections detected during pregnancy by HIV status among women screened for STI (n=358).

Infection Type	Total	HIV-infected	HIV-uninfected	p-value
	n=358	n=193	n=165	
	n (%)	n (%)	n (%)	
*Prevalent STI*	86/358 (24.0)	56/193 (29.0)	30/165 (18.2)	0.02
Chlamydia	53/358 (14.8)	33/193 (17.1)	20/165 (12.1)	0.19
Gonorrhea	17/358 (4.8)	11/193 (5.7)	6/165 (3.6)	0.36
Syphilis	2/357 (0.6)	1/192 (0.5)	1/165 (0.6)	>0.99
Trichomonas	48/320 (15.0)	29/171 (17.0)	19/149 (12.8)	0.29
More than 1 prevalent STI	29/358 (8.1)	15/193 (7.8)	14/165 (8.5)	0.81

*Incident STI*	24/358 (6.7)	21/193 (10.9)	3/165 (1.8)	<0.001
Chlamydia	15/358 (4.2)	12/193 (6.2)	3/165 (1.8)	0.04
Gonorrhea	5/358 (1.4)	4/193 (2.1)	1/165 (0.6)	0.38
Syphilis	0/358 (0)	0/193 (0)	0/165 (0)	-
Trichomonas	7/320 (2.2)	7/193 (3.6)	0	0.02
More than 1 incident STI	3/358 (0.8)	2/193 (1.0)	1/165 (0.6)	>0.99

*Other Infections/Colonization*				
Hepatitis B	10/347 (2.9)	9/183 (4.9)	1/164 (0.6)	0.02
Hepatitis C Antibody Positive	6/344 (1.7)	3/182 (1.7)	3/162 (1.9)	>0.99
Bacterial Vaginosis	10/328(3.1)	6/166 (3.6)	4/162 (2.5)	0.75
Vaginal Candidiasis	39/336 (11.6)	28/174 (16.1)	11/162 (6.8)	<0.01
Genital HSV	45/293 (15.4)	31/133 (23.3)	14/160 (8.8)	<0.001
Anogenital GBS Colonization	84/319 (26.3)	59/180 (32.8)	25/139 (18.0)	<0.01

**Table 3:** Factors associated with prevalent and incident STI in pregnancy*∗*.

	PREVALENT STI*∗∗*		INCIDENT STI *∗∗∗*	
	Unadjusted	Adjusted Odds	Unadjusted	Adjusted Odds
	Odds Ratio	Ratio	Odds Ratio	Ratio
	(95% CI)	(95% CI)	(95% CI)	(95% CI)
*HIV Status*				
Negative	Ref	Ref	Ref	Ref
Positive	**1.8 (1.1-3.0)**	**3.0 (1.4-6.4)**	**6.6 (1.9-22.5)**	**7.2 (2.1-25.0)**

Age	**0.91 (0.9-1.0)**	**0.9 (0.8-0.9)**	**0.9 (0.8-1.0)**	

*Race*				
White/Hispanic/Other	Ref	Ref	Ref	
Black	**4.7 (2.2-9.8)**	**2.7 (1.1-6.6)**	**10.1 (1.3-75.8)**	

*Marital Status*				
Married	Ref		Ref	
Single	**2.7 (1.3-5.6)**		2.0 (0.6-6.8)	

*Insurance Status*				
Private	Ref		Ref	
Government/Self-Pay	**3.1 (1.2-8.0)**		3.8 (0.5-28.7)	

History of Substance Use				
None/Marijuana	Ref		Ref	
Cocaine/IVDA/other	1.1 (0.5-2.5)		1.6 (0.4-5.9)	

Substance Use During Pregnancy				
No	Ref		Ref	
Yes	1.6 (0.5-5.5)		1.4 (0.2-11.6)	

Psychiatric Disease*∗∗∗∗*				
No	Ref		Ref	
Yes	1.4 (0.8-2.5)		**2.5 (1.1-6.0)**	

*Year of Delivery*				
2000-2006	Ref	Ref	Ref	Ref
2007-2014	**1.7 (1.1-2.8)**	**2.3 (1.1-4.7)**	**8.3 (1.9-36.0)**	**8.6 (2.0-37.8)**

*∗*STI includes chlamydia, gonorrhea, syphilis, and trichomoniasis.

*∗∗*Adjusted for all variables listed in the column for the parsimonious model.

*∗∗∗*Adjusted for HIV status, race, marital status, insurance, and year of delivery in the parsimonious model.

*∗∗∗∗*Depression, anxiety, bipolar disorder, and schizophrenia.

## Data Availability

The data used to support the findings of this study are available from the corresponding author upon request.

## Additional Points

*Brief Summary.* Pregnant women with and without HIV infection seen in our center in Alabama had high rates of STI. Maternal HIV status was independently associated with incident and prevalent STI.
